# Emerging and Versatile Non-Mammalian Model Organisms for Studying the In Vivo Antioxidant Properties of Food-Derived Bioactive Compounds

**DOI:** 10.3390/antiox14091127

**Published:** 2025-09-18

**Authors:** Alejandra Miranda-Carrazco, Verenice Torres-Salas, Rosy G. Cruz-Monterrosa, Monzerrat Rosas-Espejel, Ildefonso Guerrero-Encinas, Javier N. González-González, Luis Quihui-Cota, Andrea M. Liceaga, José E. Aguilar-Toalá

**Affiliations:** 1 Departamento de Ciencias Ambientales, División de Ciencias Biológicas y de la Salud, Universidad Autónoma Metropolitana Unidad Lerma, Av. de las Garzas 10. Col. El Panteón, Lerma de Villada 52005, Estado de México, Mexico; a.miranda@correo.ler.uam.mx; 2 Departamento de Ingeniería Agroindustrial, Universidad Autónoma Chapingo, Km 38.5 Carretera México-Texcoco, Chapingo 56230, Estado de México, Mexico; tosavere84@gmail.com; 3 Departamento de Ciencias de la Alimentación, División de Ciencias Biológicas y de la Salud, Universidad Autónoma Metropolitana Unidad Lerma, Av. de las Garzas 10. Col. El Panteón, Lerma de Villada 52005, Estado de México, Mexico; r.cruz@correo.ler.uam.mx (R.G.C.-M.); m.rosas@correo.ler.uam.mx (M.R.-E.); 4 Centro de Investigación en Alimentación y Desarrollo, A.C. (CIAD, A.C.) Carretera Gustavo Enrique Astiazarán Rosas No. 46. Col. La Victoria, Hermosillo 83304, Sonora, Mexicolquihui@ciad.mx (L.Q.-C.); 5 Protein Chemistry and Bioactive Peptides Laboratory, Department of Food Science, Purdue University, 745 Agriculture Mall, West Lafayette, IN 47907, USA

**Keywords:** unicellular microorganisms, multicellular organisms, non-mammalian model systems, antioxidant activity, oxidative stress, bioactive compounds

## Abstract

In recent years, there has been increased attention to exploring non-mammalian model organisms to study the antioxidant properties of bioactive compounds. These models include both unicellular organisms, such as *Escherichia coli* and *Saccharomyces cerevisiae,* and multicellular organisms, such as *Caenorhabditis elegans*, *Drosophila melanogaster*, and *Danio rerio*. In particular, multicellular models have emerged as promising systems due to their ease of establishing systems and maintenance, short duration of experiments, ease of genetic manipulation and genome-wide screening, availability as off-the-shelf models, safety, and cost-effectiveness. Notably, these organisms share a high degree of gene homology with humans, ranging from 65% to 84%, which positions them as powerful platforms for investigating human disease mechanisms. These advantages make them attractive candidates for investigating the potential health benefits of various bioactive compounds before resorting to mammalian models. This review delves into the rationale for utilizing these emerging non-mammalian model organisms during preliminary stages of research, emphasizing their distinct advantages over traditional mammalian models. It also highlights their significant contributions to advancing our understanding of the antioxidant mechanisms of bioactive compounds, shedding light on their potential therapeutic implications for human health. By leveraging these models, researchers can efficiently screen and validate bioactive compounds, laying a robust foundation for subsequent translational studies in mammalian systems.

## 1. Introduction

Over the last few decades, there has been increased interest regarding research on bioactive compounds, particularly those derived from food. These bioactive compounds include proteins, carbohydrates, lipids, and phenolics, among others, which are naturally present in food, thus providing health benefits beyond their nutritional value [1,2]. Bioactive compounds can also be generated during food processing, such as fermentation, or enzymatic reactions [3]. These food-derived bioactive compounds have demonstrated diverse biological properties, such as antioxidant, antimicrobial, anti-inflammatory, antitumoral, antihypertensive, antidiabetic, and antiobesity properties, among others. They have been applied not only in food for the health sector but also in cosmetic and pharmaceutical industries [4]. Among these bioactivities, particular attention has been paid to the antioxidant properties of bioactive compounds, primarily because having antioxidant activity is essential for maintaining overall health by preventing disease, delaying cell aging, and enhancing the quality and safety of food products [5,6].

In this context, the antioxidant properties of bioactive compounds are directly related to their ability to neutralize free radicals and reactive oxygen species (ROS) when these are in supraphysiological oxidative stress conditions [7]. These capabilities of neutralization are associated with the mechanisms of action involved in hydrogen atom transfer, transfer of a single electron, sequential proton loss electron transfer, and chelation of transition metals [8,9]. However, the functions of antioxidant compounds go beyond this capacity against pro-oxidant ROS, including, for example, in the modulation of cell antioxidant response, regulation of enzyme activity, repairing oxidized biomolecules, imparting immune system support, and inflammation reduction [10].

Food-derived bioactive compounds can upregulate antioxidant enzymes by inducing the expression of genes that encode these enzymes. This process is mediated by the activation of transcription factors like Nrf2 (Nuclear factor erythroid 2-related factor 2), which bind to the antioxidant response element (ARE) in the promoter regions of these genes [11]. Nrf2, encoded by the gene NFE2L2, is the master regulator of a variety of antioxidant enzymes that participate in xenobiotic metabolism, as well as in oxidative stress and inflammatory responses [12]. It has been reported that several bioactive compounds are able to activate Nrf2 by interfering with the Nrf2–Keap1 interaction, leading to the stabilization and nuclear translocation of Nrf2 [11,12]. This, in turn, upregulates the expression of antioxidant genes and the activities of corresponding enzymes, such as superoxide dismutase (SOD), catalase (CAT), glutathione S-transferase (GST), heme oxygenase 1 (HO-1), NAD(P)H quinone oxidoreductase 1 (NQO1), and glutathione peroxidase (GPx), to name a few [11,12].

The role of antioxidant compounds in repairing oxidized biomolecules entails the restoration of oxidatively damaged nucleic acids by specific enzymes (e.g., DNA glycosylases, DNA polymerases) [13], the removal of oxidized proteins by proteolytic systems (e.g., proteasomal and lysosomal) [14], and the repair of oxidized lipids by lipid-modifying enzymes (e.g., phospholipases, peroxidases or acyl transferases) [15]. Conversely, antioxidant compounds can participate in immune system support and inflammation reduction through related mechanisms. These compounds assist the function of various immune cells, such as macrophages, dendritic cells (DCs), neutrophils, T-cells, B-cells, and NK-cells, by maintaining their viability and enhancing their ability to respond to pathogens [16]. In this context, these types of bioactive compounds with antioxidant properties can protect immune cells from damage caused by oxidative distress, maintaining the cellular integrity and function of these cells, and support the proliferation of immune cells [17]. In a similar way, antioxidants can modify the balance between pro- and anti-inflammatory cytokines by increasing the release of anti-inflammatory cytokines with simultaneous inhibition of the pro-inflammatory cytokines or through one of these aforementioned mechanisms [18].

Based on the above, the benefits obtained are numerous and highly significant for human health; thus, it is important to use appropriate methods to evaluate the antioxidant properties of bioactive compounds. The successful evaluation of bioactive compounds with antioxidant properties depends on using reliable assays to screen for antioxidant activity [10,19,20]. Given that the evaluation of antioxidant activity from bioactive compounds has evolved significantly over time, the first methods were based in chemical (in vitro) assays, followed by biomolecule-based and cell-based (in situ) assays, and, finally, animal (in vivo) studies. At present, novel methods and strategies have been in development in order to evaluate antioxidant activity, including the use of advanced spectroscopic and imaging techniques, genomic and proteomics approaches, in silico/bioinformatics methods, and emerging in vivo models using uni- and multicellular organisms. Therefore, this review aims to provide an in-depth exploration of the main emerging model organisms used for evaluating the antioxidant activity of bioactive compounds while emphasizing in vivo model systems using unicellular and non-mammalian multicellular organisms.

## 2. Conventional Methods and Models for Evaluating Antioxidant Activity

### 2.1. Chemical In Vitro Methods

To date, several antioxidant methods have been developed, mainly based on chemical mechanisms, such as electron transfer, hydrogen transfer, or chelation of transition metals. The most commonly used methods today include 2,2-diphenyl-1-picrylhydrazyl (DPPH), 2,2′-azino-bis-(3-ethylbenzothiazoline-6-sulfonic acid) (ABTS), ferric reducing antioxidant power (FRAP), and oxygen radical absorbance capacity (ORAC), among others [21,22].

**Figure 1:**
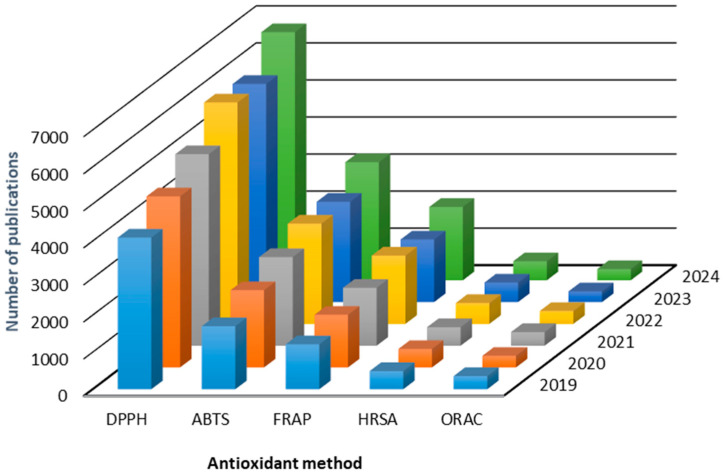
Comparative chart of recent publications related to the use of chemical in vitro methods for evaluating antioxidant activity. The compilation of the data was performed on the Web of Science Core Collection database (Clarivate analytics, USA) on 31 May 2025. Original scientific studies (excluding review articles, book chapters) dating from January 2019 to January 2025 were included. The main search terms used were the “name” of the antioxidant method (e.g., DPPH or 2,2-diphenyl-1-picrylhydrazyl), consulting scientific studies published in English. DPPH: 2,2-diphenyl-1-picrylhydrazyl; ABTS: 2,2-azinobis-3-ethylbenzothiozoline-6-sulphonate; FRAP: ferric ion reducing antioxidant parameter; HRSA: hydroxyl radical scavenging activity; ORAC: oxygen radical absorbance capacity.

The frequency of use of antioxidant methods is highlighted in Figure 1, which showed the literature accumulating (ca. 62,000 publications) from January 2019 to January 2025 related to the use of various antioxidant methods. From this analysis, the most used method was DPPH, with a total of 32,445 publications, followed by the ABTS method with 14,783 publications and, to a lesser extent (<10,000 publications), the FRAP, HRSA, and ORAC methods. Others methods, such as CUPRAC (cupric reducing antioxidant capacity) and TRAP (total reactive antioxidant potential), were included in the analysis but excluded from the comparative chart as they had less than 200 publications over the time period used in the analysis.

These chemical methods present several advantages in their applicability. For example, they are quick and easy to implement, offer a certain degree of precision and reproducibility, and are adaptable and flexible for evaluating different types of bioactive compounds and samples [23]. Finally, they allow for a quantitative evaluation of antioxidant activity/capacity in numerical terms, which can be compared and analyzed statistically [21,22]. In particular, the most popular antioxidant method, DPPH (Figure 1), is inexpensive, and its preparation as a radical does not require additional steps [24]. Thus, its implementation is simple and quick compared to other in vitro chemical methods. The above can partially explain why DPPH is the most used (reported) in vitro chemical method for evaluating antioxidant activity.

In contrast, some disadvantages associated with these chemical methods are related to the fact that their evaluation does not fully reflect the antioxidant activity in a complex biological system [10,19]. Due to the nature of the assays, they can only provide information about one form of antioxidant activity related to the ability to scavenge reactive species or its reducing power [22]. It has also been reported that these methods can exhibit interferences, as they are generally spectrophotometric methods, and compounds present in complex matrix samples interfere with the reaction reading. Furthermore, these methods do not consider the synergistic interactions between different antioxidants present in complex matrix samples, thus evaluating antioxidant activity individually. Lastly, these methods do not reflect aspects of bioaccessibility and bioavailability of the antioxidant compounds in a biological context [25]. In summary, chemical (in vitro) antioxidant methods are valuable due to their sensitivity and specificity for reactive species, particularly free radicals, as well as their speed and simplicity of application. However, almost all chemical methods have limitations, and it is important to consider complementing these methods with biological studies to obtain a comprehensive evaluation of antioxidant activity in a physiological context.

### 2.2. Computational In Silico Models

Advances in computational chemistry programs and the availability of open databases allow for in silico prediction methods to study the structure, function, and properties of molecules, including their antioxidant activity, in a more cost-effective and efficient manner than traditional laboratory methods [26,27]. These computational tools enable the evaluation of large datasets of molecules, which saves costs and reduces the need for laboratory animals. These methods are often seen as environmentally friendly; however, little attention has been given to the significant energy demands of maintaining cloud-based databases, running molecular simulations, and performing other computational tasks that might take several days [28]. It is essential to recognize the environmental impact of computational chemistry, assess existing approaches, and prioritize those with the least detrimental environmental effects.

Given the chemical diversity of antioxidants, which include vitamins, proteins (i.e., enzymes) and peptides, carotenoids, hormones, minerals, saponins, steroids, and glutathione, among others [29], multiple computational strategies have been developed. Currently, the most widely used methods include QSAR (quantitative structure–activity relationships), molecular docking predictions of bioactivity, and ADME (absorption, distribution, metabolism, and excretion) [26,27,30]. These approaches allow for rapid virtual screening of molecules with potential antioxidant activity and provide insights into their behavior under different physicochemical conditions. However, predicting activity in complex biological systems remains challenging. To address this, recent advances in artificial intelligence (AI) and machine learning have enabled the development of algorithms that capture complex structure–function/activity relationships and simulate biological processes more accurately [31].

QSAR is a computational modeling approach that aims to establish quantitative links between the molecular structure of a substance and its biological activity properties [32]. The principle of QSAR states that compounds with similar structures tend to have similar biological activities [33]. Therefore, it is necessary to understand the biological activity of molecules with similar functional groups [34]. This technique helps to initially identify chemical entities with potential antioxidant properties, thereby reducing the number of candidate compounds that require experimental validation and supporting the characterization of new molecules when experimental information is limited [32]. Nevertheless, QSAR has its limitations; for example, it does not consider the effects of experimental variables, such as light exposure, temperature, and reaction time, on molecular responses. It also does not account for biological aspects, such as the bioavailability and metabolic processing of the compounds being evaluated [30].

Alternatively, one of the most effective tools being used for evaluating antioxidant activity is molecular docking. This computational technique reveals how a molecule interacts with potential targets based on their structure and chemical properties [35]. Many candidate antioxidants, especially new molecular entities, are initially assessed using this method [36,37,38,39]. Several factors can be analyzed through molecular docking, including binding affinity, conformational stability, hydrophobicity, and binding areas [26,35]. This method is often used in conjunction with in vitro chemical methods to assess the antioxidant activity of new molecules, particularly proteins [40,41,42]. The most common protein in molecular docking evaluations of potential antioxidants is Keap1 [27], which regulates the Nrf2 antioxidant pathway [43].

Once the structure is understood, predictions and validations of bioactivity enable the rapid virtual screening of potential antioxidant compounds by examining their similarity to substances previously documented in databases. There are software programs that predict multiple trials. For example, PeptideRanker is a web server that estimates the biological activity of peptides based on the similarity of their amino acid sequences to those of known antioxidants [44]. This software has been widely used not only for potential antioxidants but also for various types of drugs, such as those related to obesity [45], diabetes [46,47], allergies [48], antimicrobial applications [49], and hormonal treatments [50]. However, some caveats include the threshold selection (e.g., the lower the threshold, the higher the risk of false positives; the higher the threshold, the higher the risk of reducing the true positive rate) and peptide modifications (e.g., acetylation of the N-terminus, the amidation of the C-terminus) that can influence the detected bioactivity.

CPPpred is another web server designed to predict the likelihood of a peptide being cell-penetrating [51]. Additionally, ADME analysis is a crucial pharmacokinetic assessment in the development of drugs and other bioactive substances. These studies are typically conducted on organic xenobiotics tested as potential drug candidates. This process helps evaluate how a compound interacts with the human body, providing insights into its effectiveness, safety, and potential toxicity before clinical testing in living organisms [52]. Predicting the ADME properties of potential antioxidants is essential to ensure they fit the criteria for viable drug candidates.

Overall, the improvements in sequencing resources and increased computational power have enabled the development of in silico methods for studying antioxidant molecules prior to in vivo analyses. Simulating the molecule’s structure allows researchers to predict bioactive trials and analyze its potential behavior in the body [53,54,55]. In silico analyses do not replace in vitro and in vivo tests but provide complementary, valuable information to guide future experimentation.

### 2.3. Conventional In Vivo Models

In vivo models have been used to evaluate the antioxidant effect of various bioactive compounds. Animal models, primarily rats and mice, have been widely used in these studies. Additionally, human/clinical trials, both with pre-existing pathologies and clinically healthy individuals, have been utilized. Various studies conducted using in vivo models have demonstrated an inverse relationship between the consumption of foods rich in antioxidant bioactive compounds and the development/progression of various diseases, a finding that has also been confirmed in epidemiological studies [22,25,56].

Despite the obvious advantages of implementing these models for evaluating antioxidant activity, it is important to mention that they also have some drawbacks. For example, when using complex living organisms, there is a wide variety of interactions, which can make it difficult to isolate and fully understand the specific effect of an antioxidant, especially if a mechanistic study is required [22,57]. There has also been variability among the animals and human subjects due to individual differences in them, such as age, sex, genetics, and health status that are sometimes difficult to control. Similarly, multiple external factors can influence the antioxidant response in human studies, including diet, lifestyle, and the environment [25,58]. In addition, experimental procedures in living organisms can cause stress and alterations, which can influence the results of the studies. Finally, one major drawback of these in vivo studies is that they can be costly and logistically complicated to carry out, especially when dealing with more complex or long-lived organisms. In vivo experiments using mammalian animals can also raise ethical issues and require approval from ethics committees and compliance with local and international regulations and laws [25,59].

Despite these disadvantages, in vivo studies remain a crucial part of scientific research and are essential for understanding how antioxidants interact in a real biological system. As an intermediate stage, in the search for better models to evaluate the antioxidant activity of bioactive compounds, some assays that utilize isolated cells (e.g., erythrocytes) [60] and tissues (e.g., cell lines like Caco-2) [61] have been proposed. However, they also present some limitations. For example, these two models are specific to human body cells, so they do not reflect the complexity of a complete biological system [60,61].

## 3. Emerging Models for Evaluating the Antioxidant Activity of Bioactive Compounds

In recent years, new models have emerged to evaluate the antioxidant activity of bioactive compounds. These include models using unicellular microorganisms and model systems with non-mammalian multicellular organisms [62,63,64]. The classification of these emerging models is divided into two main groups: (1) model systems of unicellular microorganisms (e.g., *Saccharomyces cerevisiae* and *Escherichia coli*) and (2) model systems of non-mammalian multicellular organisms (e.g., *Caenorhabditis elegans*, *Drosophila melanogaster*, and *Danio rerio*).

### 3.1. Model Systems Using Unicellular Microorganisms

Unicellular microorganism models, such as *Saccharomyces cerevisiae* and *Escherichia coli*, are simple organisms composed of a single cell. They are valuable for molecular biology and genetics studies because the complete genome of these yeasts [62] and bacteria [65] has been sequenced and published, and the genome sequence data are easily searchable and accessible in numerous databases, as well as due to their ease of manipulation and rapid reproduction. In the field of antioxidant activity research, these models offer ease of cultivation and manipulation, making them cost-effective to maintain and efficient for conducting experiments [66,67,68]. As already mentioned, model systems of unicellular microorganisms are considered a superior alternative over chemical in vitro methods for screening antioxidant compounds, primarily because these models allow for understanding the cellular response to oxidative stress conditions as well as the function of antioxidant defense systems [62]. In particular, having detailed knowledge of its biology allows for the identification and study of specific genes related to antioxidant activity, which in turn will serve to investigate how genes and metabolic pathways are involved in the antioxidant response [69,70,71].

Given that they are unicellular microorganisms, the microbial culture conditions and experiments can be controlled with great precision, which facilitates the reproducibility of the results, providing high consistency as an antioxidant model [64,72]. For example, studies indicate that *Saccharomyces cerevisiae* and *Escherichia coli* were useful for evaluating the antioxidant properties of bioactive compounds. Gao, Fang, Wang, Lan, Wang, Du, Guan, Liu, Brennan, Guo, Brennan, and Zhao [66] assessed the antioxidant properties of a flavanol glycoside named hyperoside (quercetin-3-*O*-galactoside) on *Saccharomyces cerevisiae* with hydrogen-peroxide-, carbon-tetrachloride-, or cadmium-induced oxidative stress. Overall, the authors found that this bioactive compound was able to significantly increase cell viability, decrease lipid peroxidation, and lower intracellular ROS. Similarly, Kavitake, Veerabhadrappa, Sudharshan, Kandasamy, Devi, Dyavaiah, and Shetty [67] found that galactan exopolysaccharide derived from *Weissella confuse* KR780676 was capable of reducing the intracellular ROS generated in a *Saccharomyces cerevisiae* antioxidant-deficient strain (yeast mutant model) with oxidative stress generated by hydrogen peroxide (H_2_O_2_). Additionally, this bioactive compound increased the viability (10–15%) of yeast mutant cells, enhancing their resistance to oxidative stress and preventing aging. On the other hand, the protective effect of essential oils (rosemary *(Rosmarinus officinalis*), thyme (*Thymus vulgaris*), oregano (*Origanum compactum* Benth.), eucalyptus (*Eucalyptus globulus* Labill.), and basil (*Ocimum basilicum* L.) towards oxidative stress induced by H_2_O_2_ in *Saccharomyces cerevisiae* was investigated by Ridaoui, Guenaou, Taouam, Cherki, Bourhim, Elamrani, and Kabine [70]. In that study, it was reported that several essential oils (i.e., basil, oregano and thyme) showed the best antioxidant activity in yeast cells by decreasing lipid peroxidation and protein carbonylation as well as restoring the activity of antioxidant (CAT, SOD, and GR) and metabolic enzymes (succinate dehydrogenase) against H_2_O_2_-induced oxidative stress. Likewise, Piechowiak and Balawejder [72] reported that onion (*Allium cepa* L.) skin extract exhibited an antioxidant effect towards cadmium-induced oxidative stress in *Saccharomyces cerevisiae* by decreasing the generation of ROS and lipid peroxidation. Bruna-García, Isabel-Redondo, Sabater-Munoz, and Miguel-Castro [69] assessed the antioxidant properties of fat essences obtained from Iberian dry-cured ham using the yeast *Saccharomyces cerevisiae* as an experimental model with oxidative stress induced by H_2_O_2_. The authors measured the antioxidant capacity through yeast growth parameters and survival rate and found that the fat essences (rich in monounsaturated fatty acids) were able to recover the growth rate and increase yeast survival.

The bacteria *Escherichia coli* has also been used as an experimental model for evaluating the antioxidant activity of bioactive compounds. Zhou, Wang, Yang, Liu, and Zhang [64] used a microbial test system based on *Escherichia coli* for evaluating the antioxidant capacity of phytochemicals, plant extracts, fruits, and vegetables. In most cases, the samples tested were able to increase the cell growth rate (between two- and four-fold) under oxidative stress (H_2_O_2_) conditions. Similarly, Wu, Yu, Zhang, Dong, Li, and Wang [71] observed that plant extracts from hardy rubber tree (*Eucommia ulmoides* Oliv.) were able to extend the growth rate of *Escherichia coli* cells under H_2_O_2_-induced oxidative stress. In particular, the extract from the leaf portion of the tree was generally stronger than the extract obtained from the bark.

Table 1 shows that unicellular microorganism models have been used to evaluate the antioxidant activity of a wide variety of bioactive compounds derived from food. The most commonly used experimental model for oxidative stress conditions was induced with hydrogen peroxide, given its accessibility and ability to produce hydroxyl free radicals that are among the most physiologically important. The main parameters evaluated for antioxidant activity included cellular growth, survival rate, lipid peroxidation, and ROS generation. In this context, the ability of a bioactive compound to protect cells from oxidative stress can have a direct impact on the cell growth and survival of these microorganisms. In summary, both *Saccharomyces cerevisiae* and *Escherichia coli* offer valuable insights into the antioxidant activity of bioactive compounds, but their respective biological complexities and sensitivities can influence the outcomes and applicability of the results. Overall, it was observed that in the period of analysis of this review, *Saccharomyces cerevisiae* was widely used, while *Escherichia coli* was studied to a lesser extent. Yeast strains, such as *Saccharomyces cerevisiae,* offer versatility in inducing various oxidative stress conditions, significant antioxidant responses, and applicability towards diverse bioactive compounds. On the other hand, *Escherichia coli* exhibits a growth rate increase under oxidative stress and broad applicability across diverse compounds, allowing for potency comparisons of different plant extracts. Overall, these microbial unicellular models are convenient and robust for evaluating antioxidant activity due to their ease of manipulation, detailed knowledge of their biology, well-characterized genomes, and ability to represent fundamental biological processes.

### 3.2. Model Systems Using Non-Mammalian Multicellular Organisms

*Caenorhabditis elegans*, *Drosophila melanogaster*, and *Danio rerio* are examples of model systems of non-mammalian organisms composed of multiple cells commonly used for in vivo model systems. Although more complex than unicellular microorganisms, these systems offer advantages for broader studies related to development, physiology, and genetics [73]. In particular, these organisms possess between 65% and 84% of gene homology for the study of human diseases [74], which allows researchers to study the functions of human genes and their roles in diseases within these simpler organisms. Thus, being multicellular models, they allow for the study of antioxidant effects in a more complex biological context compared to studies with unicellular models [75,76,77]. These models also have a short lifecycle, which facilitates conducting studies quickly and efficiently. Similarly to unicellular models, these multicellular models have a well-documented genome, which makes it easier to study genes related to antioxidant responses. In this regard, these organisms retain many biological pathways as mammals, which facilitates the extrapolation of results to other organisms, including humans [63,78,79]. Additionally, while assessing the antioxidant activity of a bioactive compound, the potential side effects (e.g., toxicity) of the evaluated compounds can also be evaluated. Moreover, they are commonly used models for longevity and aging studies, allowing for assessing the impact of antioxidants on these processes [80,81].

In terms of assay development, multicellular organism models offer some methodological advantages over unicellular models. For example, the availability of fluorescent markers and genetic manipulation techniques allows for the detailed study of cellular and molecular processes during the antioxidant response [77,82]. Furthermore, in early developmental stages, these organisms are transparent, facilitating the observation and study of biological processes, such as redox, in real time [78,83]. Moreover, they lend themselves to high-throughput assays to evaluate the antioxidant activity of a large number of bioactive compounds in a short period of time [84,85].

Table 1 shows some of the studies that have implemented *Caenorhabditis elegans* as an experimental model for evaluating the antioxidant properties of bioactive compounds. For instance, Wu, et al. [86] evaluated the antioxidant activity of organic sulfides derived from fresh long-stamen onion (*Allium macrostenom*) by using this nematode. The authors found that these bioactive compounds enhanced the activities of the antioxidant enzymes (superoxide dismutase, catalase, and glutathione peroxidase), improved motility, and extended the lifespan of the nematodes under paraquat-induced oxidative stress conditions. In another study, González-Paramás, Brighenti, Bertoni, Marcelloni, Ayuda-Durán, González-Manzano, Pellati, and Santos-Buelga [76] evaluated the antioxidant activity of an anthocyanin-rich extract from bilberry (*Vaccinium myrtillus* L.) using *C. elegans* exposed to thermal-induced oxidative stress. They observed that the treatment with 5 μg/mL anthocyanin extract showed protective antioxidant effects towards the accumulation of ROS and increased thermal resistance in *C. elegans*, both in stressed and non-stressed young and aged worms. Other studies showed that different concentrations of bioactive peptides (ranging from 0.125 to 3.0 mg/mL) obtained from canary seed (*Phalaris canariensis*) and edible cricket (*Acheta domesticus*) protein increased the survival rate of *C. elegans* under both acute (tBOOH) and chronic (paraquat) oxidative stress conditions. These bioactive peptides also decreased the generation of ROS and significantly upregulated the antioxidant-related gene gst-4 [63,87,88].

**Figure 2:**
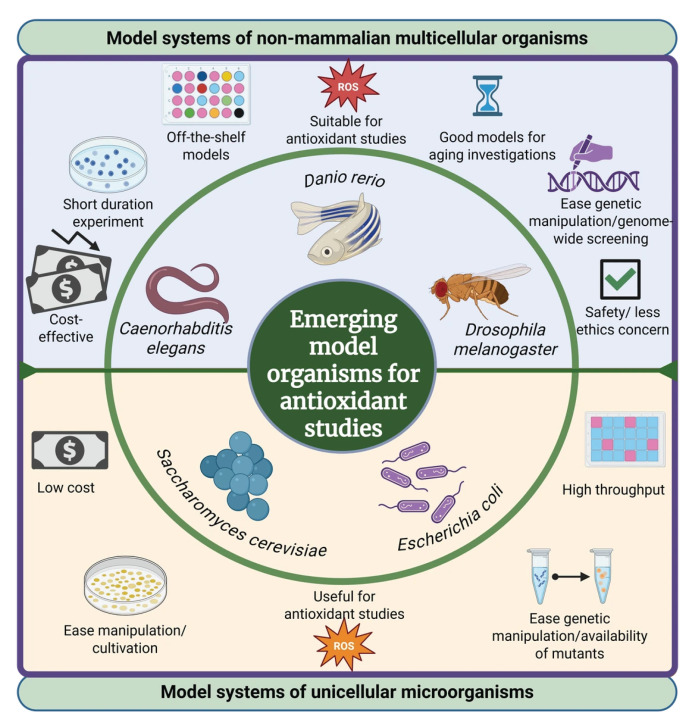
Characteristic features of multicellular and unicellular microorganisms as emerging models for antioxidant studies (created using Biorender.com).

In Figure 2, we can observe the main characteristics and/or features of different multicellular and unicellular microorganisms that have been used for evaluating antioxidant properties of food-derived bioactive compounds. Overall, the use of both multicellular and unicellular model organisms is crucial in evaluating the antioxidant activity of bioactive compounds. In this sense, multicellular models provide insights into complex biological interactions and systemic effects. These models allow for the observation of antioxidant effects in the context of a whole organism, including developmental, neurological, and aging processes

As indicated in the previous section, unicellular models offer a simple yet powerful platform for initial screenings and mechanistic studies due to their ease of genetic manipulation, rapid growth, and suitability for high-throughput assays, making them ideal for investigating cellular responses to oxidative stress. Similarly, multicellular models, where hydrogen peroxide is commonly used to induce oxidative stress because of its accessibility and ability to generate physiologically relevant hydroxyl radicals, allow for comparable evaluations. Together, these models provide a comprehensive approach to understanding the antioxidant properties of bioactive compounds by enabling the assessment of cellular toxicity, reactive species production, antioxidant enzyme activity, gene expression, lipid peroxidation, protein carbonylation, glutathione levels, lifespan (anti-aging) effects, and parameters related to cellular growth and survival. These parameters have allowed for the evaluation of the effect of antioxidant compounds on complex biological systems, providing a more comprehensive assessment and understanding of the antioxidant activity of a bioactive compound. This integrative strategy contributes significantly to elucidating molecular mechanisms, evaluating therapeutic potential, and determining safety and efficacy profiles in the context of oxidative-stress-related diseases.

In this context, the evaluation of cellular toxicity by the bioactive compound being studied is considered the first and foremost critical step in assessing its potential therapeutic effect [89]. This initial step ensures the compound’s safety by identifying any harmful effects it may exert on cells, such as inducing apoptosis, necrosis, or other forms of cytotoxicity. Cellular toxicity refers to the detrimental effects a bioactive compound can have on living cells, and its evaluation involves exposing cells to varying concentrations of the compound to determine its cytotoxic threshold. Common assessments include cell viability assays, such as the MTT or XTT assay, which measure mitochondrial activity as an indicator of cell health. Additionally, advanced techniques like flow cytometry and staining methods, such as propidium iodide or Annexin V, are employed to differentiate between apoptotic and necrotic cells, providing a comprehensive understanding of the compound’s impact on cellular systems [90].

On the other hand, many studies have evaluated the production of reactive species/molecules (e.g., ROS and reactive nitrogen species [RNS]) that can induce oxidative stress when produced in excess. Then, bioactive compounds may decrease the ROS and RNS levels. The production of these reactive molecules is often measured using fluorescent probes like 2,7-Dichlorofluorescein diacetate (DCFH-DA), which detects intracellular ROS; this analysis reveals the compound’s ability to act as an antioxidant under specific conditions.

**Figure 3:**
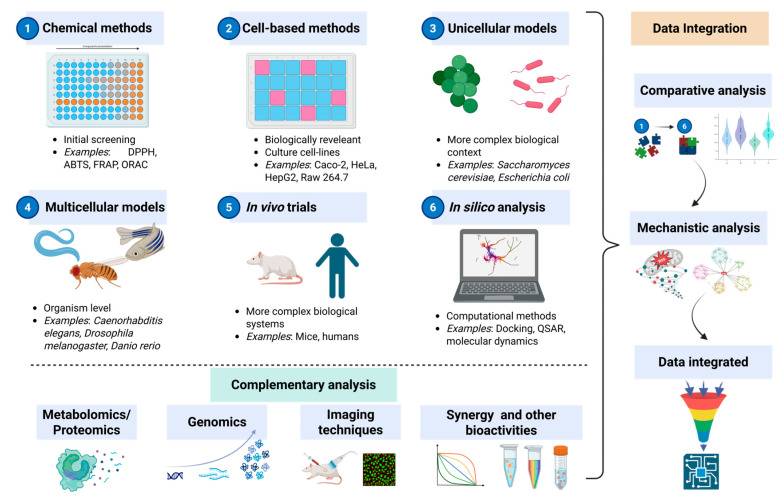
Proposed integrated workflow for the assessment of antioxidant activity of bioactive compounds using chemical (in vitro) and cell-based (in situ) methods, in vivo models/trials, and in silico analysis (created using Biorender.com).

In summary, evaluation of the antioxidant activity of bioactive compounds is crucial for understanding their potential health benefits, especially in the context of disease prevention and management. Figure 3 shows a comprehensive workflow that incorporates various approaches, including in vitro chemical methods, cell-based assays, emerging models using unicellular and multicellular organisms, and in silico analysis, increasing the complexity of methods and their biological importance. In addition, several other strategies were included as complementary analyses that can be employed to gain a deeper understanding of their mechanisms of action, efficacy, and overall potential health benefits. It should be noted that this workflow is a proposal in which one or more steps can be omitted or followed in different orders according to the aims of each study related to the antioxidant properties of bioactive compounds.

**Table 1:** Selected studies using emerging in vivo models for evaluating the antioxidant activity of bioactive compounds.

**Unicellular Microorganism Used**	**Experimental Model for Oxidative Stress**	**Bioactive Compounds**	**Evaluation, Biomarkers, and/or Use of the Model**	**References**
*Saccharomyces cerevisiae*	Hydrogen peroxide, cadmium, and carbon tetrachloride	Hyperoside (quercetin-3-O-galactoside), galactan (microbial exo-polysaccharide), plant essential oils, onion extract (*Allium cepa* L.), Iberian ham extracts rich in fatty acids and polyphenols	Cellular toxicity of the bioactive compound Levels of reactive species production Lifespan study (anti-aging) Activity of antioxidant enzymes (SOD, CAT, GP, and GR) Degree of lipid peroxidation and carbonylated proteins Level of the glutathione molecule Study of cellular growth and survival parameters	[66,67,69,70,72]
*Escherichia coli*	Hydrogen peroxide	Fruit, vegetable, and plant extracts (*Eucommia ulmoides* Oliv., *Potentilla fruticosa* y *Ginkgo biloba*)	Study of cellular growth parameters Activity of antioxidant enzymes (SOD, CAT, GP, and POD) Analysis of expression of antioxidant genes (CAT and SOD)	[64,68,71]
**Multicellular Organism Used**	**Experimental Model for Oxidative Stress**	**Bioactive Compounds**	**Evaluation, Biomarkers, and/or Use of the Model**	**References**
*Caenorhabditis elegans*	Thermal treatment (>35 °C, <5 h), paraquat (herbicide), hydrogen peroxide, tert-Butyl hydroperoxide	Extracts of blueberry (*Vaccinium myrtillus* L.), chive (*Allium macrostemon* Bunge), red algae (*Agarophyton chilense*), and herb *Artemisia argyi*; bioactive peptides derived from edible crickets (*Gryllodes sigillatus*); fermented beverage based on kiwi and pitahaya juices; canary seed (*Phalaris canariensis* L.) peptides	Study of growth parameters of the organism Levels of reactive species production Antioxidant enzyme activity (SOD, CAT, and GP) Study of lifespan (anti-aging) Study of survival (mobility) Levels of carbonylated proteins Analysis of antioxidant gene expression (CAT and SOD) Degree of lipid peroxidation	[76,81,82,84,86,87,88]
*Drosophila melanogaster*	Hydrogen peroxide, paraquat (herbicide), heat stress (>30 °C), high-sugar diet	Hemp seed oil (*Cannabis sativa* L.), fucoidan polysaccharide (*Sargassum fusiforme*), blueberry extracts (*Vaccinium myrtillus* L.) and amaranth leaf extracts (*Amaranthus dubius*), bioactive peptides derived from rice	Glutathione level Lipid peroxidation degree Antioxidant enzyme activity (SOD and CAT) Survival study (mortality) Lifespan study (anti-aging) Oxidative stress index (trehalose levels in hemolymph)	[77,79,80,83,91]
*Danio rerio*	Dimethyl phthalate, copper sulfate, scopolamine	Ginger roots (*Zingiber officinale* Roscoe), rice bran oil, mango peel extract (*Mangifera indica* cv. Ataulfo), essential oil of *Angelica purpurascens* Gilli., apple cider vinegar	Analysis of antioxidant gene expression (CAT, SOD, GP, and GR) Levels of reactive species production Antioxidant enzyme activity (SOD, GP, and CAT) Lipid peroxidation degree Survival study (mortality and survival) Study of organism growth parameters	[75,85,92,93]

SOD: superoxide dismutase; CAT: catalase; GR: glutathione reductase; GP: glutathione peroxidase; POD: peroxidase.

## 4. Conclusions

The application of non-mammalian organisms to study in vivo antioxidant properties of bioactive compounds is gaining interest among the research community. This is particularly due to the lower cost involved in the use of these organisms, as well as the fact that it is considered more ethically acceptable to use such organisms to gather preliminary data.

Unicellular microorganism models are ideal for research at the cellular and molecular levels, while non-mammalian multicellular organisms are more suitable for studies at a broader (full organism) systematic level that includes more complex biological processes. In particular, *Caenorhabditis elegans*, *Drosophila melanogaster*, and *Danio rerio* are valuable models for research in the field of antioxidants and oxidative stress conditions, offering a wide range of advantages in terms of genetic simplicity, fast lifecycle, and biological relevance. It is important to keep in mind that these models also have some limitations. For example, these organisms are easily prone to contamination from the media being used, airborne particles, etc. Therefore, careful handling and strict precautions must be implemented when handling organisms and the experimental materials. Although multicellular organisms can have a relatively high homology to human genes, they cannot model all processes in the human body. Despite their genetic similarities, they could still have metabolic and physiological process variations that limit their application to studying a broad range of mammalian metabolisms.

Overall, both model systems are suitable as emerging models for evaluating the antioxidant activity of bioactive compounds. However, it is advisable to use a combination of several models to evaluate the antioxidant activity of a specific compound, from in vitro antioxidant methods, especially for mechanistic antioxidant studies, to in vivo animal and clinical assays to consider the parameters of bioaccessibility and bioavailability of the evaluated compounds and to obtain a holistic understanding of the antioxidant effect. Additionally, it is suggested to complement mechanistic studies with in silico evaluations (e.g., molecular docking and molecular dynamics simulations) to observe the interaction between antioxidant bioactive compounds and specific reactive species. This approach can provide a more comprehensive assessment and understanding of the antioxidant activity of a bioactive compound.

## Data Availability

Not applicable.
